# Orofacial Myofunctional Therapy in Obstructive Sleep Apnea Syndrome: A Pathophysiological Perspective

**DOI:** 10.3390/medicina57040323

**Published:** 2021-04-01

**Authors:** Venkata Koka, Andrea De Vito, Gabriel Roisman, Michel Petitjean, Giulio Romano Filograna Pignatelli, Davide Padovani, Winfried Randerath

**Affiliations:** 1 Department of Sleep Medicine, Hospital Antoine Beclere, 92140 Clamart, France; gabriel.roisman@aphp.fr (G.R.); michel.petitjean@aphp.fr (M.P.); 2 Ear Nose Throat (ENT) Unit, Head & Neck Department, Santa Maria delle Croci Hospital, Romagna Health Service, 48121 Ravenna, Italy; giulioromano.filogranapignatelli@auslromagna.it (G.R.F.P.); davide.padovani@auslromagna.it (D.P.); 3 Clinic of Pneumology and Allergology, Center for Sleep Medicine and Respiratory Care, Institute for Pneumology at the University Witten/Herdecke, 42699 Solingen, Germany; randerath@klinik-bethanien.de

**Keywords:** sleep-disordered breathing, apnea, myofunctional therapy, oropharyngeal exercises, oral motor exercises

## Abstract

Obstructive sleep apnea (OSA) syndrome is a multi-factorial disorder. Recently identified pathophysiological contributing factors include airway collapsibility, poor pharyngeal muscle responsiveness, a low arousal threshold, and a high loop gain. Understanding the pathophysiology is of pivotal importance to select the most effective treatment option. It is well documented that conventional treatments (continuous positive airway pressure (CPAP), upper airway surgery, and dental appliance) may not always be successful in the presence of non-anatomical traits, especially in mild to moderate OSA. Orofacial myofunctional therapy (OMT) consists of isotonic and isometric exercises targeted to oral and oropharyngeal structures, with the aim of increasing muscle tone, endurance, and coordinated movements of pharyngeal and peripharyngeal muscles. Recent studies have demonstrated the efficacy of OMT in reducing snoring, apnea–hypopnea index, and daytime sleepiness, and improving oxygen saturations and sleep quality. Myofunctional therapy helps to reposition the tongue, improve nasal breathing, and increase muscle tone in pediatric and adult OSA patients. Studies have shown that OMT prevents residual OSA in children after adenotonsillectomy and helps adherence in CPAP-treated OSA patients. Randomized multi-institutional studies will be necessary in the future to determine the effectiveness of OMT in a single or combined modality targeted approach in the treatment of OSA. In this narrative review, we present up-to-date literature data, focusing on the role of OSA pathophysiology concepts concerning pharyngeal anatomical collapsibility and muscle responsiveness, underlying the response to OMT in OSA patients.

## 1. Introduction

Obstructive sleep apnea (OSA) is an increasingly common form of sleep-disordered breathing (SDB), with an incidence of 15% in men and 5% in women in adult age and characterized by repetitive collapse or obstruction of the pharyngeal airway during sleep. According to the Wisconsin sleep cohort study in the United States, the estimated prevalence of moderate to severe OSA increased by 14% to 55% over the past two decades [1]. Obesity is a high-risk factor, with an incidence of OSA in 50% of the obese population. Aging is also an independent risk factor, with 50% of elderly men having a respiratory disturbance index (RDI) above 13/h [2]. The clinical picture of OSA may include one or more symptoms, including snoring, nocturnal polyuria, excessive daytime sleepiness, morning headache, fatigue, neurocognitive deficits, personality alterations, reduced libido, irritability, depressive symptoms, and anxiety [3]. Excessive daytime sleepiness is frequent and increases the risk of vehicle crashes and occupational accidents [4].

OSA is a multi-factorial disorder, where anatomical and non-anatomical factors can contribute to determine different pathophysiological traits [5,6,7,8]. Hence, understanding the pathophysiology of OSA is of pivotal importance for choosing the most effective therapy.

Continuous positive airway pressure (CPAP) is mainly directed at impaired anatomy as a first-line therapy, however, 50% of CPAP users become non-adherent over time and some refuse the treatment [9]. Alternative non-CPAP treatments, such as oral appliances, upper airway (UA) surgery, and weight loss, may result in unpredictable results if administered without consideration of non-anatomical phenotypes.

Orofacial myofunctional therapy (OMT) has been recently introduced as an OSA treatment option and consists of isotonic and isometric exercises targeted to oral and oropharyngeal structures, with the aim of increasing muscle tone, endurance, and coordinated movements of pharyngeal and peripharyngeal muscles.

In this narrative review, we present up-to-date literature data, focusing on the role of OSA pathophysiology concepts concerning pharyngeal anatomical collapsibility and muscle responsiveness when considering OMT in OSA patients.

## 2. Materials and Methods

A narrative review was prepared using MEDLINE, The Cochrane Library, and EMBASE, in accordance with the following criteria: “obstructive sleep apnea”, “OSA pathophysiology”, and “OSA treatment” for selection of articles between 1985 to 2020. We focused our selection criteria mainly on original articles, evaluating the entirety of information and concepts expressed to collect the most important and thorough aspects of up-to-date myofunctional therapy applied to adult OSA patients, focusing on the role of up-to-date OSA pathophysiology concepts concerning pharyngeal anatomical collapsibility and muscle responsiveness.

## 3. Discussion

OSA is defined as a multi-factorial disorder, characterized by four contributing phenotypes: airway collapsibility, poor pharyngeal muscle responsiveness, a low arousal threshold (AT), which contributes to unstable ventilatory control, and a high loop gain (LG) or a hypersensitive ventilatory control feedback loop [6,7,8,9] (Table 1). The arousal threshold (AT) is the level of ventilatory drive contributing to arousal from sleep. A lower AT does not allow enough time for respiratory stimuli to recruit the pharyngeal muscles and reopen the airway before arousal and destabilizes breathing. Loop gain measures ventilatory control system sensitivity and is defined as the ratio of the ventilatory response to reduction in ventilation. The components of steady-state LG include both “plant gain” (i.e., lung volume, gas exchange rate, circulatory delay), which relates to a change in end-tidal CO_2_ for a given change in ventilation, and a “controller gain”, which relates to ventilatory sensitivity to CO_2_; an overall LG of less than 1 is typical of a stable respiratory control system [8,9]. Given that airway obstruction in OSA only occurs during sleep, the combination of an anatomical predisposition combined with sleep state-dependent changes in non-anatomical contributors is crucial in driving OSA [10,11,12].

### 3.1. Upper Airway Collapsibility

Upper airway collapsibility or impaired anatomy is the major driving force in the pathogenesis of OSA. Passive UA collapsibility is increased in obesity via deposition of adipose tissue in regions surrounding the airway, including within the soft tissues of the neck and pharyngeal muscles. Increased neck circumference was reported to be significantly related to the severity of OSA [13]. Other risk factors include increased tongue volume and tongue fat [14], central adiposity [15], narrowing of craniofacial structures [16], and rostral fluid shifts [17].

Even though imaging methods, such as conventional craniofacial radiography, computerized tomography (CT), and magnetic resonance imaging (MRI), reveal static anatomy during wakefulness, the functional or dynamic status of the pharyngeal airway cannot be inferred by such measurements, as UA collapsibility is sleep stage, body position dependent, and influenced by the interaction of non-anatomic factors [18].

Functional anatomy or passive UA collapsibility is measured in the laboratory as Pcrit (critical closing pressure) using CPAP dial ups and downs during NREM sleep. The critical closing pressure, or Pcrit, is the luminal pressure at which the UA collapses [19,20]. On average, OSA subjects have a higher Pcrit than those without OSA [6,21,22].

### 3.2. Muscle Responsiveness and Effectiveness

The human pharynx is a predominant soft tissue structure and collapsible segment with varying intraluminal pressures, depending on the dynamic balance of intraluminal pressure and neural drive to the UA dilator muscles [23]. The genioglossus muscle, the largest and phasic UA dilator muscle, receives neural drive from six pattern generator neurons from the brain stem, respiratory drive reflex inputs from pressure sensitive mechanoreceptors in the pharynx, and chemical drive from increases in CO_2_ and hypoxia. Remaining muscles are predominantly tonic and are most active during wakefulness. Genioglossus muscle activity is sleep stage dependent and decreases from N3 to N2 to REM sleep stages. EMG activity of genioglossus increases in relation to increasing ventilatory drive, except for in one-third of OSA patients who show minimal or absent muscle activation [6], resulting in a low muscle responsiveness to neural drive to airway narrowing during sleep. In some OSA patients, UA dilatation may not be achieved despite robust genioglossus muscle activation, ultimately terminating in arousal.

Various causative factors have been attributed to OSA, such as muscle fiber change from aerobic type I to anaerobic type 2 with less endurance [24,25], muscle fiber loss and muscle atrophy [26], altered fiber orientation [27], and neuronal degeneration [25].

Tagged MRI revealed counterproductive or bidirectional movements characterized by anterior motion at the base of the tongue followed by airway narrowing at the level of the soft palate, especially in mild to moderate OSA, and little to no movement during inspiration in severe OSA [28]. This may result from a maladaptation in the co-activation of different muscle compartments.

### 3.3. Phenotype Targeted Treatment

In 2013, Eckert et al. [6] developed the PALM Classification in order to report all four main pathophysiological factors (Pcrit, AT, LG, and Muscle responsiveness) in a unique scale in order to identify the OSA patients who are most likely to require CPAP (23%) and those who can be treated with non-CPAP therapies (77%).

The passive Pcrit scale identifies the severity of UA impairment: Pcrit above +2 cmH_2_O (PALM 1) with severe UA collapsibility; Pcrit between +2 and −2 cmH_2_O (PALM 2) with moderate UA collapsibility; and Pcrit < −2 cmH_2_O (PALM 3) with minor UA collapsibility (Table 2).

If Pcrit is above +2 cmH_2_O (PALM 1), collapsibility is significantly related to the apnea–hypopnea index (AHI), as collapsibility is the major driving cause for OSA. PALM 2 may include normal subjects, as well as patients with mild to severe OSA. Eckert et al. observed 66% of PALM 2 patients and 100% of PALM 3 patients as having moderate or minor UA collapsibility, respectively, with one or more non-anatomical traits, emphasizing the need to phenotype traits in order to successfully target non-CPAP treatments in such patients.

The identification of physiological OSA traits by CPAP dial ups and dial downs is invasive and requires skilled personnel and specially equipped sleep laboratories. Landry et al. [29] demonstrated that therapeutic CPAP levels ≤ 8 cmH_2_O predict mild airway collapsibility (Pcrit < −2 cmH_2_O), with a sensitivity of 89% and specificity of 84%, and suggested that CPAP pressures could be accurately used in differentiating mild from severely collapsible airway to advocate non-anatomical treatments. OSA patients with mild collapsibility were shown to respond well to oral appliances and non-anatomical treatments.

Recently, a clinical score was developed by Genta et al. [30] to discriminate pharyngeal collapsibility in men using anthropometric and polysomnographic indices. The presence of NREM- Obstructive Apnea Index/AHI > 0.44 yields 2 points, and waist circumference > 106 cm, mean obstructive apnea duration > 22.1 s, and REM-AHI > 39.9 yield 1 point each to predict a Pcrit > 2.5 cmH_2_O (Area Under Curve = 0.96). A score ≥ 3 showed a sensitivity of 90.9% and a specificity of 84.3%. A score < 3 may help to identify OSA patients with low Pcrit who may respond to non–CPAP therapies.

Edwards et al. [31] developed a clinical score to predict low AT from a sleep diagnostic study, allocating a score of 1 to each of three criteria, the nadir SpO_2_ > 82.5%, fraction of hypopneas > 58.3%, and AHI < 30/h. A total score of 2 predicted low AT, with a sensitivity of 80.4% and specificity of 88%. Eckert et al. [32] reported that increasing AT with eszopiclone can reduce OSA severity, particularly if patients have a low initial AT. Eszopiclone increased AT by 30%, with a 43% reduction of AHI in patients with low AT. This opens a perspective for new combined non-CPAP treatment, targeting multiple non-anatomical traits.

Terrill et al. [33] applied a mathematical chemoreceptor control model to calculate ventilatory drive non-invasively using polysomnographic variables, measuring loop gain at different cycle frequencies; they reported that OSA patients with high loop gain can be successfully treated with acetazolamide. OSA patients with good muscle compensation and high LG responded well with supplemental oxygen [34]. Such patients with a high LG may not respond to other conventional non-CPAP treatments, as it has been well documented that high loop gain predicts the failure of mandibular advancement devices and UA surgery [35,36].

Recently, Messineo et al. [37] reported that a simple breath-holding test in office settings can estimate LG contribution to OSA by measuring ventilation after a 20-s breath-hold and maximum breath-hold duration. The predictors for high LG were maximum breath-hold duration and second breath response. Spontaneous maximum breath-hold duration was 38 ± 3.4 s in controls compared to 28.8 ± 2.7 s in OSA patients. Loop gain correlated inversely with maximum breath-hold duration. This simple test for identifying high loop gain may help to predict the success or failure of non-CPAP treatment before undertaking sleep tests in labs.

Therefore, both clinical and diagnostic PSG findings can help identify non-anatomic phenotype traits in clinical setting when selecting a single or a combined approach on an individual basis.

### 3.4. Treatments to Improve Muscle Function

In recent years, many successful attempts have been made to improve genioglossus muscle function during sleep by direct hypoglossal nerve stimulation. Hypoglossal nerve stimulation by implantable device to enable phasic stimulation was used, with a reduction of AHI in two-thirds of OSA patients [38]. This STAR trial revealed 33% of subjects as non-responders. Moreover, the procedure is invasive and yields unpredictable results, and failures have been related to the presence of high passive collapsibility (Pcrit) and a high LG [39].

Pharmacotherapy for OSA has been applied in an attempt to increase muscle responsiveness; hypnotic drugs, such as zolpidem (GABAergic), have been shown to increase pharyngeal muscle activity during airway narrowing in OSA patients [37]. In a recent randomized double-blinded study, a combination of atomoxetine and oxybutynin was shown to reduce AHI by 63% and increase genioglossus muscle activity 3-fold in 20 patients [40]. This trial is very promising as an alternative method to conventional treatments in the presence of mild UA collapsibility and no cardiovascular morbidity.

### 3.5. Orofacial Myofunctional Therapy (OMT)

OMT offers good potential for the treatment of OSA as an alternative method for increasing muscle tone in a noninvasive manner.

First described in 1918 by Roger [41] for proper tongue positioning in the oral cavity in order to improve mandibular growth, nasal breathing, and facial appearance, OMT was proposed as a treatment of OSA by Guimaraes [42] to improve UA dilator function. Puhan et al. reported that UA muscle training, by means of didgeridoo playing, significantly reduced OSA severity [43].

Guilleminault et al. [44] reported that abnormal oropharyngeal development is associated with sensory changes in the tongue and apraxia, which in turn potentiates further maladaptation of the palate and jaw, as well as hypotonia of pharyngeal dilators, and predisposes to upper airway collapsibility. The authors stated that impairment of lingual gnosis and praxis may persist in adulthood and advocated early intervention in childhood.

OMT consisted of isotonic and isometric exercises targeted to oral and oropharyngeal structures including lips, tongue, and the soft palate, facial muscle exercises, as well as stomatognathic functions, including suction, breathing, speech, swallowing, and chewing, with the aim of increasing pharyngeal and peripharyngeal muscle tone, endurance, and coordinated movements of tongue.

A randomized control study of oropharyngeal exercises on 31 patients with moderate OSA conducted by Guimaraes et al. [45] revealed a significant reduction in neck circumference and OSA severity. Ten out of fifteen (62.5%) patients shifted from moderate to mild OSA and wot patients to no OSA after 3 months of OMT. The duration of exercises of 3 to 6 months was considered to allow an adequate remodeling of UA muscles and improve success.

Similar results were reported by Verma et al. [46] in a study on 20 mild to moderate OSA patients treated with oropharyngeal exercises over a 3-month period. They reported a significant reduction in neck circumference (38.4 ± 1.3 cm to 37.8 ± 1.6 cm).

Weight reduction was shown to reduce tongue fat, tongue volume, and AHI in obese OSA patients [47]. The mechanism of action of OMT in the reduction of tongue fat and neck circumference was presumed to be the result of direct training of the genioglossus and pharyngeal muscles [48] and the restructuring or remodeling of the airway [45].

Systemic review studies in the past have confirmed the benefits of OMT in terms of improvements in snoring, sleep quality, lowest oxygen saturation, daytime sleepiness, and reduction of AHI [48,49]. A meta-analysis by Camacho et al. [50] of nine studies consisting of 120 patients confirmed the efficacy of OMT in the treatment of OSA, with a 50% improvement rate in AHI (24.5 ± 14.3 to 12.3 ± 11.8 events/h), non-significant improvements in the average lowest SaO_2_ (83.9 ± 6.0 to 86.6 ± 7.3%), significant improvements in reported snoring and Epworth sleepiness scale (ESS). A study by Baz et al. [51] reported a 10% reduction of snoring from pre- to post-OMT.

The decrease in snoring and daytime sleepiness may result from improvements in muscle responsiveness, UA muscle gain, and subsequent reductions of inspiratory flow limitations and arousals. Stomatognathic exercises also enable the coordinated recruitment of different compartments of tongue and other pharyngeal muscles and probably eliminate bidirectional tongue movements typical of OSA. OMT may also improve the coordinated action of pharyngeal and peripharyngeal muscles, improve chewing, speech, breathing, and swallowing functions in OSA patients, thus improving quality of life.

Guimaraes et al. [45] observed a significant reduction of AHI in REM stage. Therefore, OMT may provide a sustained increase of pharyngeal dilator tone, especially of the genioglossus in all stages of sleep. OMT may also be used in REM-predominant OSA, which is known to cause substantial metabolic [52] and cardiovascular risks [53].

OMT has been employed with conventional treatments such as mandibular devises, surgery, and CPAP for moderate to severe OSA in the adult and pediatric population, suggesting the need to improve muscle responsiveness to obtain long-term results.

Guilleminault et al. [54] retrospectively analyzed the efficacy of post-operative OMT, applied to 24 children treated (AHI: 0.4 ± 0.3) with adenotonsillectomy and palatal expansion. This study included 11 children who received OMT post-operatively compared to 13 children who did not receive OMT (controls). After a 4-year follow-up, the control group showed a recurrence of OSA (AHI: 5.3 ± 1.5/h), whereas the post-operative OMT group remained cured with no recurrence. This highlights that the removal of obstruction does not necessarily increase muscle responsiveness and underlines the potential application of OMT in a multi-modality approach. OMT helps in the restoration of nasal breathing and reduced mouth breathing, which in turn improves nocturnal breathing and reduces OSA in both adult and pediatric populations [54,55]. Moreover, Villa et al. [55] observed a significant increase in the objective measurements of tongue strength, tongue peak pressure, and endurance.

Chuang et al. [56] conducted a comparative cohort study with a passive myofunctional therapy, using an oral device with built-in tongue bead for one year in children with OSA and reported a significant improvement in nasal breathing during sleep, mandible linear growth, airway antero-posterior distance, and quality of life. The tongue bead in the oral device may function like a foreign object, which targets the swallowing reflex and increases tongue movement, mimicking myofunctional therapy. AHI changed from 3.75 ± 2.48 to 2.16 ± 1.80 in the treatment group, compared to 3.09 ± 2.55 to 3.95 ± 3.74 in the control group. Mandibular advancement to over 50% reduces the passive collapsibility trait and the tongue bead may improve the muscle responsiveness trait.

Suzuki et al. [57] reported the efficacy of OMT for middle-aged and elderly OSA patients treated with CPAP and reported a discontinuation in 50% of moderate OSA patients. The patients performed MFT three times a day at home for 6 months in parallel with CPAP treatment during sleep. Forty-five percent of patients with severe OSA improved to moderate status, and 54% of patients with moderate OSA improved to mild OSA.

There is a growing interest in identifying phenotyping tools and the site of obstruction at clinical settings in order to select targeted OMT exercises. O’Connor-Reina et al. [58] evaluated the Iowa Oral Performance Instrument (IOPI) to aid in the identification of the obstruction site in OSA patients. The authors observed a significant correlation of IOPI tongue pressures and VOTE classification (T size) during drug-induced sleep endoscopy (0 = no obstruction, 1 = partial obstruction, 2 = complete obstruction). IOPI tongue and lip measurements were significantly lower than standard values in OSA patients, and the authors suggested that the presence of high values of tongue pressures may exclude tongue site obstruction.

The main drawback of OMT is non-adherence. However, adherence and the effectiveness of OMT can be improved by frequent interventions by personnel, education, visual coaching, and smart phone health apps. Kim et al. [59] developed and evaluated a myofunctional therapy support program (MTSP) based on the self-efficacy theory and observed a significant increase in the rate of adherence. During the 12-week program, the MTSP group received two 30-min face-to-face education sessions, eight mobile text messages, and a weekly 10-min telephone coaching session. The rate of adherence to exercises according to daily exercise logs was 82.06 ± 23.70% in the MTSP group (*n* = 15) and 72.52 ± 30.09% in the control group (*n* = 15).

O’Connor-Reina et al. [60], who conducted a randomized controlled trial on patients with severe OSA (AHI > 30) with exercises through interactions with a smartphone app reported a high adherence in the app group of 90% compared to 50% in the control group who did not have app interaction. Increased adherence may be due to ease of contact with health professionals, as well as acoustic and visual feedback on patient performance, provided by the app.

In a recent study, Diaféria et al. [61] compared the results of the treatment of 100 OSA patients with a placebo, placebo vs. OMT, CPAP therapy, and combined CPAP associated with OMT and reported how adherence to CPAP increased when CPAP was associated with OMT. The adherence to treatment was 30% in the CPAP group and 65% in the CPAP with OMT group. The hours of CPAP use at the third month follow-up was significantly higher in the CPAP with OMT group (5.1 ± 2.3 h/day) compared to the CPAP alone group (3.6 ± 1.8 h/day). The increased adherence in the CPAP and OMT combined group might have been biased by frequent monitoring, which may have improved CPAP adherence. However, no change was noted in therapeutic pressures in both groups. OMT helps to reposition the tongue, improves nasal breathing, and reduces mouth leaks in patients with CPAP.

None of the studies reported in literature showed that OMT used alone decreased the AHI to <5/h (primary goal of OSA treatment) when applied in adult patients with moderate to severe OSA. When applied with conventional treatments in moderate to severe OSA, OMT improves success rates as well as treatment adherence. OMT may be tried as a first-line treatment in mild OSA patients who may present mild obstruction combined with multiple non-anatomical OSA traits.

## 4. Conclusions

OSA pathophysiology is rather complex, with manifold combinations of anatomical and non-anatomical predominant factors that can be differentiated by means of clinical and polysomnographic data. Current therapy available to OSA patients is oriented around targeted treatment options with the aim of achieving the best outcomes. OMT can play a central role in OSA treatment due to its proven effects on the UA muscular framework. OMT may be applied as a stand-alone treatment both in adult and pediatric OSA patients, with efficacy reported by literature data. Recent studies also suggest the potential for OMT as part of a combined approach for improving pharyngeal muscle function. Randomized multi-institutional studies are necessary in order to define adequate protocols for OMT as a stand-alone therapy in mild OSA or in a combined modality approach with other conventional and/or non-anatomic treatments.

## Figures and Tables

**Table 1:** Pathophysiological obstructive sleep apnea (OSA) traits.

**Impaired anatomy:**
Upper airway collapsibility
**Non-anatomical traits:**
Poor muscle responsiveness
Low arousal threshold
High loop gain

**Table 2:** PALM Scale.

**PALM 1** (23% of OSA patients)
Severe upper airway collapsibility (Pcrit > +2 cmH_2_O)
**PALM 2** (58% of OSA patients)
Moderate upper airway collapsibility (Pcrit −2 to +2 cmH_2_O)
**PALM 3** (19% of OSA patients)
Mild upper airway collapsibility (Pcrit < −2 cmH_2_O)

Note: P: critical closing pressure; A: arousal threshold; L: loop gain; M: muscle responsiveness.

## Data Availability

Not applicable.
